# (Non-)translational medicine: targeting bacterial RNA

**DOI:** 10.3389/fgene.2013.00230

**Published:** 2013-11-08

**Authors:** Adam M. Dinan, Brendan J. Loftus

**Affiliations:** School of Medicine and Medical Science, Conway Institute of Biomolecular and Biomedical Research, University College DublinDublin, Ireland

**Keywords:** antisense, non-coding RNA, small RNA, riboswitch, antibiotics, bacteria, external guide sequence, locked nucleic acids

## Abstract

The rise and spread of antibiotic resistance is among the most severe challenges facing modern medicine. Despite this fact, attempts to develop novel classes of antibiotic have been largely unsuccessful. The traditional mechanisms by which antibiotics work are subject to relatively rapid bacterial resistance via mutation, and hence have a limited period of efficacy. One promising strategy to ameliorate this problem is to shift from the use of chemical compounds targeting protein structures and processes to a new era of RNA-based therapeutics. RNA-mediated regulation (riboregulation) has evolved naturally in bacteria and is therefore a highly efficient means by which gene expression can be manipulated. Here, we describe recent advances toward the development of effective anti-bacterial therapies, which operate through various strategies centered on RNA.

## RNA-BASED REGULATION IN BACTERIA: OF NATURAL IMPORTANCE

**FIGURE 1:**
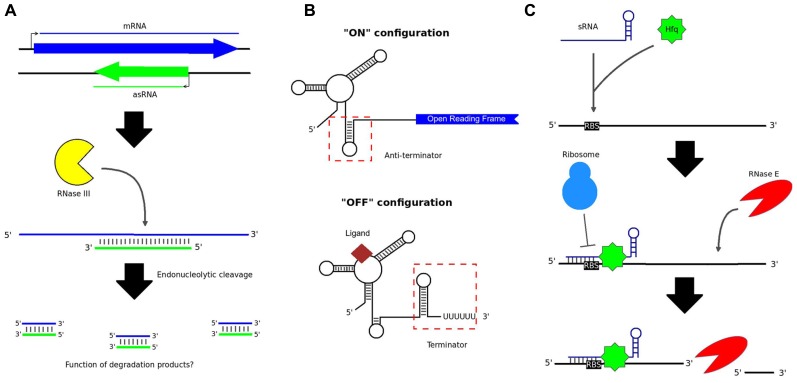
**Various types of non-coding RNA in bacteria.** **(A)** asRNAs are transcribed from the opposite strand to protein-coding genes. The resulting dsRNA structure can serve as a substrate for cleavage by RNase III; however, it is not known whether the short degradation products resulting from this process have any cellular function. **(B)** Riboswitches control the expression of downstream genes by folding to form either terminator or anti-terminator hairpin loops, depending on the presence or absence of an appropriate signal ligand. **(C)** Intergenic sRNAs typically work in combination with the Hfq chaperone protein, binding the RBS of target mRNA transcripts and preventing translation of the mRNA. RNase E cleavage of the mRNA may subsequently occur.

Non-coding RNAs (ncRNAs) occur naturally in bacteria and can function as regulators of gene expression. ncRNAs may be transcribed either in-*cis*, i.e., from the same genomic loci as their targets, or in-*trans*, from discrete loci (Waters and Storz, 2009). A major class of *cis-*encoded ncRNAs, known as antisense RNAs (asRNAs), originate from the opposite strand to overlapping protein-coding genes (Thomason and Storz, 2010). An asRNA can occur over a portion of the opposite gene or over the entire length of the gene (Thomason and Storz, 2010). This leads to the formation of double-stranded RNA (dsRNA) molecules, which can present as targets for enzymatic digestion, thereby resulting in decreased translation of the mRNA (**Figure 1A**; Lasa et al., 2012). Whole transcriptome analysis, in particular the advent of RNA sequencing (RNA-seq), has revealed that anywhere from 13 to 49% of genes in bacteria may be subject to some degree of antisense regulation (Lasa et al., 2012).

Another class of *cis*-acting**regulatory RNAs originate from the 5′ untranslated regions (UTRs) of genes and can cause premature transcriptional termination (Lai, 2003). Riboswitches are prominent members of this class (Lai, 2003). Riboswitches generally control the transcription of downstream protein-coding genes by folding alternately to form either terminator or anti-terminator hairpin loops (**Figure 1B**; Serganov and Nudler, 2013). The highly structured aptameric region of a riboswitch binds selectively to a small ligand – such as an amino acid, an enzyme cofactor, or an ion – when the ligand is present in the cell (Mironov et al., 2002; Nahvi et al., 2002). The structure of the region adjacent to the aptamer, known as the expression platform, is then altered, dictating whether or not transcription can proceed (Serganov and Nudler, 2013).

*Trans*-acting RNAs include intergenic small RNAs (sRNAs). In contrast to asRNAs, which generally bind targets over large portions of their lengths, most known sRNAs bind to target mRNAs via short (7–12 nt) stretches, known as *seed regions* (Storz et al., 2011). The binding site is generally overlapping with, or in close proximity to, the ribosome binding site (RBS) of the target mRNA, thereby occluding 70S ribosome formation and translation initiation (**Figure 1C**; Waters and Storz, 2009). Endonucleolytic cleavage of the mRNA may also occur (Caron et al., 2010), perhaps to increase the speed of gene silencing, or to render silencing irreversible. In many lineages, the interaction is facilitated by the Hfq chaperone protein (Vogel and Luisi, 2011). However, in species lacking Hfq, other features of an sRNA, such as its GC-content, may be important for target recognition (Arnvig and Young, 2012).

## HIJACKING NATURAL DESIGNS – ARTIFICIAL ANTISENSE AND sRNAs

Antisense RNAs complementary to custom mRNA sequences were first designed in *Escherichia coli *in the late 1990s (Engdahl et al., 1997), and the technique has since been extended to gram-positive species (Ji et al., 2004). Expressed antisense technology has been used to target a range of bacterial genes, including those involved in DNA exchange (Wang and Kuramitsu, 2005), central metabolism (Greenberg et al., 2010), and antibiotic resistance (Ramirez et al., 2013). The antisense molecule is typically complementary to the RBS of the target mRNA, to facilitate steric block of translation initiation (Woodford and Wareham, 2009). A positive correlation between the length of an asRNA and the degree of target gene regulation has been reported for *E. coli* (Tatout et al., 1998). However, structural features of the target mRNA are an important consideration in the design process, given that interaction sites must be accessible to the antisense transcript (Deere et al., 2005).

More recently, artificial *trans*-encoded sRNAs (atsRNAs) directed at custom mRNAs have also been developed (Man et al., 2011). These atsRNAs consist of three separate domains – a seed region, a Hfq binding site, and a rho-independent terminator (RIT) – and are able to repress the expression of both endogenous and exogenous target genes in *E. coli *(Man et al., 2011). Gene silencing by atsRNAs is more efficient than can generally be achieved with antisense strategies (Man et al., 2011), and is most potent when the seed region is present within a single-stranded part of the molecule (Park et al., 2013). Engineering of atsRNA constructs against particular targets is possible, and has been used to produce transcripts directed at *E. coli *outer membrane porin and flagellin genes (Sharma et al., 2011).

## RIBOSWITCHES AND LIGAND ANALOGS

A number of antibacterial compounds whose mode of action was initially unclear are now known to act through riboswitches (Blount and Breaker, 2006). For example, L-aminoethylcysteine (AEC) is a lysine analog that inhibits the growth of several gram-positive bacterial species (McCord et al., 1957). However, it has only recently become apparent that its mechanism of action involves binding to a lysine riboswitch and causing premature transcriptional termination of essential anabolic genes (Blount et al., 2007). Specifically designed analogs have proven effective at killing bacteria by binding to riboswitches *in vitro *(Blount et al., 2007; Kim et al., 2009) and in reducing pathogenicity in animal infection models (Mulhbacher et al., 2010).

Enthusiasm for the utility of ligand analogs as a novel drug class has been tempered somewhat by the potential for unintended off-target effects. For example, the riboflavin analog roseoflavin inhibits the growth of *Listeria monocytogenes* by switching the FMN riboswitch to an “off” configuration (Mansjö and Johansson, 2011). However, roseoflavin also increases the expression of certain virulence genes in the process, perhaps by interacting with riboflavin metabolism enzymes in the cell (Mansjö and Johansson, 2011). Knowledge of potential off-target binding partners is an important consideration in drug development. Some riboswitches are known to employ slightly different binding mechanisms for a given ligand than do proteins (Blount and Breaker, 2006), and such mechanistic differences should be considered in the design process.

Recent advances have resulted in a scaling up of high-throughput screens for RNA structures and small molecules that interact with one another. The method of Tran and Disney (2012), for example, allows the screening of over three million combinations of RNA aptamers and molecules to find interacting pairs. Strategies to monitor the activity of riboswitches in the presence of novel ligands are also available; for example, a screening method involving molecular beacon probes has been developed using an unmodified version of the adenine riboswitch (Chinnappan et al., 2013). This approach can, in principle, be applied to any class of riboswitch and occurs within the native transcriptional context.

## TYPE II CRISPR SYSTEMS

**FIGURE 2:**
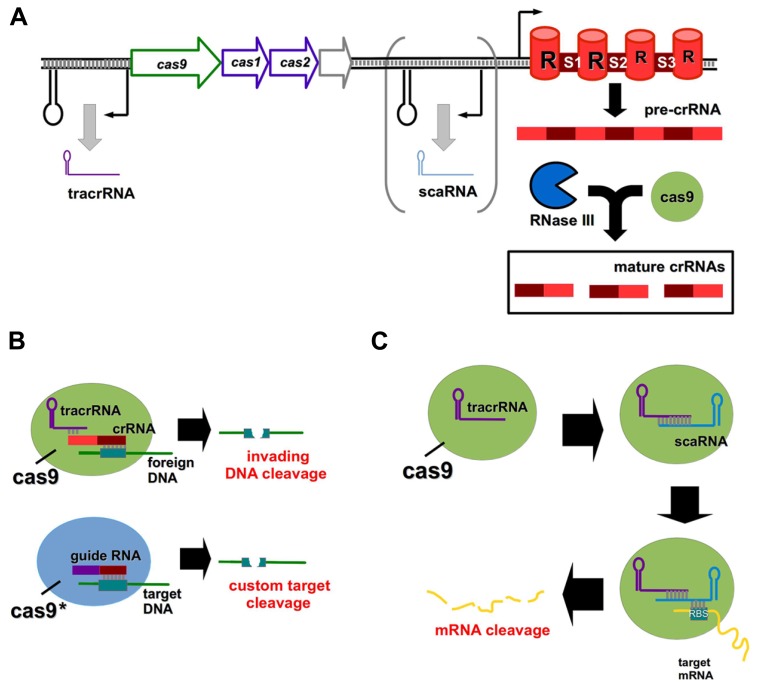
**CRISPR/Cas systems as genome editing tools and regulators of gene expression.** **(A)** General outline of a type II CRISPR/Cas system; the scaRNA component is present in certain lineages only. Pre-crRNA is processed by the combined action of Cas9 and RNase III to form mature crRNAs, each containing a repeat (R) element and a spacer (S1–S3) region. **(B)** Cas9 normally functions to cleave viral or plasmid DNA in the bacterial cell upon association of the mature crRNA with a complementary foreign DNA molecule. The tracrRNA and crRNA components can be replaced by a guide RNA, and the Cas9 enzyme may be mutated to achieve custom DNA target cleavage. **(C)** The novel scaRNA of certain type II CRISPR systems mediates Cas9 cleavage of a target mRNA transcript by associating with the mRNA at its RBS.

Clustered regularly interspaced short palindromic repeats (CRISPR) and CRISPR-associated (Cas) systems are bacterial defense mechanisms, which can cleave invading DNA from plasmids and bacteriophages (Sorek et al., 2013). There are three primary CRISPR types (I–III) found in bacteria, differing from one another in Cas protein composition and mechanism of action (Sorek et al., 2013). All CRISPR systems function by the incorporation of short (∽30 nt) stretches of invading nucleic acids into so-called spacer regions within the CRISPR array (Barrangou et al., 2007). Transcription from the array gives rise to a precursor CRISPR RNA (pre-crRNA), which is processed into mature crRNA fragments, each comprised of a spacer and a repeat region (**Figure 2A**; Horvath and Barrangou, 2010). The spacer of a crRNA binds specifically to a complementary site known as a proto-spacer in the target DNA to facilitate cleavage (Gasiunas et al., 2012).

While type I and type III systems utilize multiple Cas proteins for target degradation, type II systems require only the Cas9 endonuclease (Chylinski et al., 2013), and have thus received much attention for their potential use as genome editing tools (Horvath and Barrangou, 2013). The formation of mature crRNAs in type II systems involves a *trans*-activating crRNA (tracrRNA) and the activity of the endonuclease RNase III (Deltcheva et al., 2011). Engineered type II CRISPR systems can be introduced directly to bacterial cells on plasmids to induce a variety of DNA mutations (Jiang et al., 2013). For human genome editing, a codon-optimized version of Cas9 has been developed which contains a nuclear localization signal to ensure correct compartmentalization (Cong et al., 2013). The tracrRNA and crRNA components may be fused to create custom guide RNA molecules (**Figure 2B**; Cong et al., 2013; Mali et al., 2013), and multiple custom spacers can be incorporated into a single CRISPR array to induce discrete target cleavage (Cong et al., 2013).

It was thought that CRISPR systems were capable of targeting only invading DNA, however a recent study has shown that a small, CRISPR/Cas-associated RNA (scaRNA) mediates endogenous gene regulation in *Francisella novicida* by mRNA destabilization (**Figure 2C**; Sampson et al., 2013). This scaRNA is part of the type II CRISPR system of *F. novicida*, and base pairs with both the tracrRNA and the RBS of the target mRNA (Sampson et al., 2013). scaRNAs are predicted to occur in a number of other important pathogens, including *Neisseria meningitidis* and* Campylobacter jejuni *(Sampson et al., 2013). Rational manipulation of the scaRNA component of this system may enable selective gene regulation in both prokaryotic and eukaryotic systems in future.

## REPURPOSING RNase P

An ingenious yet mechanistically simple mode of RNA-based gene regulation has been devised which utilizes the intrinsic activity of the ribozyme RNase P. RNase P is an evolutionarily ancient and highly conserved endonuclease which normally functions in bacteria to cleave precursor tRNA (ptRNA) molecules at their 5’ ends (Kazantsev and Pace, 2006). Short oligonucleotides known as external guide sequences (EGSs) can be designed such that they bind to target mRNA molecules, resulting in a structure which resembles a ptRNA and is cleaved by RNase P (Li et al., 1992).

Furthermore, EGSs can be induced from bacterial plasmids to inhibit gene expression (Guerrier-Takada et al., 1995), including the expression of genes for antibiotic resistance (Soler Bistué et al., 2009). Multiple EGSs can be targeted toward essential genes and act in an additive manner to reduce bacterial viability (McKinney et al., 2001). Significantly, EGS-mediated gene repression functions in the presence of up to three mismatches along a 15 nt stretch, implying that several point mutations of the target would be required for the evolution of bacterial resistance (McKinney et al., 2001). Currently, the identification of suitable mRNA-EGS interaction sites is laborious, for example through randomization of EGS sequences and subsequent selection of target regions (Lundblad et al., 2008). However, advances toward the rational computational prediction and design of ribozyme splice sites (Meluzzi et al., 2012) may help to ameliorate this difficulty.

## DELIVERY MECHANISMS AND CONSIDERATIONS

The treatment of pathogenic infection is predicated on the delivery of drug compounds to the site of infection in the body and into the bacterial cell. Natural RNA is susceptible to nucleolytic attack prior to cell entry. To circumvent this fact, synthetic oligonucleotides have been developed, including peptide nucleic acids (PNAs), which are modified to contain a peptide backbone (Good et al., 2001); and DNA mimics known as phosphorodiamidate morpholino oligomers (PMOs; Geller et al., 2003). These compounds offer considerable increases in extra-cellular stability; however, major obstacles remain in permeating the bacterial membrane (Good et al., 2000; Geller et al., 2003).

Uptake efficiency may be increased by the conjugation of PNAs and PMOs to short cationic peptides (Nikravesh et al., 2007; Mellbye et al., 2009). These positively charged molecules likely function by co-localising the synthetic oligonucleotides with the negatively charged bacterial outer membrane. Peptide conjugates have been developed to act as conventional asRNAs (Deere et al., 2005) and also to act as EGSs (Lundblad and Altman, 2010). The utility of antisense peptide conjugates *in vivo *has been demonstrated using mouse models of *E. coli *infection (Tilley et al., 2007). Thermoresponsive hydrogels, which are formulated as liquids and harden at mammalian body temperature, have recently been used to deliver peptide-PMOs to mouse wounds, improving healing by targeting the *Staphylococcus aureus* gyrA mRNA (Sawyer et al., 2013).

A relatively underexplored strategy is to synthesize oligonucleotides as locked nucleic acids (LNAs). LNAs are inherently more stable molecules than naturally occurring RNA molecules, because they are “locked” into a 3′-*endo* conformation (Koshkin et al., 1998). LNA/DNA hybrid oligomers that contain a stretch of at least six DNA bases can serve as substrates for RNase H cleavage to enhance target downregulation (Braasch and Corey, 2002). These hybrid molecules have been shown to effectively function as EGSs to decrease amakicin resistance in *E. coli*, and were found to be more efficient at gene silencing than PMOs (Soler Bistué et al., 2009; for a comparison of these methods, see **Table 1**). Unlike the synthetic compounds described above, LNAs carry a negative charge, which means that they cannot easily be conjugated with peptides. However, it has recently been shown that LNA/DNA oligomers are naturally uptaken by *E. coli* cells at a higher rate than regular nucleic acids (Traglia et al., 2012). Additional research will need to be carried out on methods to further increase the level of uptake (which is at a modest 14%), however this finding offers promise for the future utility of LNA technology in combating infection.

**Table 1:** Comparison of different forms of synthetic nucleic acids used in therapeutic strategies that target bacterial RNAs.

	PNA	PMO	LNA/DNA oligomers
Nuclease resistance	High	High	High
RNA binding strength relative to nucleic acids	Increased	Increased	Increased
Typical delivery method	Conjugation to peptide	Conjugation to peptide or direct modification	Naturally uptaken
Toxicity	Low^*^	Low^*^	Low
Electric charge	Non-ionic	Non-ionic	Anionic
Target specificity	Moderate	Moderate	High
Induction of RNase H cleavage	No	No	Yes

* Note: peptide conjugate may be toxic.

## BACTERIAL RESISTANCE TO RNA-BASED STRATEGIES

Reports of bacterial resistance to peptide-based delivery strategies have been published (Ghosal et al., 2012; Puckett et al., 2012). Certain peptide-PNA conjugates are transported across the *E. coli* cell membrane by the SmbA transporter, with the PNA component being the substrate (Ghosal et al., 2012), and mutations to SbmA can prevent efficient uptake (Ghosal et al., 2012; Puckett et al., 2012). Alternative transporters are known to be available, and screening of antisense PNAs on *ΔsbmA *strains has been successful (Ghosal et al., 2012). Notably, however, this mechanism of resistance relates strictly to the mode of transport used to induce cellular uptake, and is distinct from the gene regulation induced by the antisense molecules themselves. Resistance via mutation of target mRNA molecules has not been documented, perhaps indicating that sequence alterations to regulatory regions such as RBSs, which are generally targeted, are likely to be very rare.

Bacterial resistance to riboswitch ligand analogs is also known. For example, pyrithiamine is an antibacterial substance which acts by mimicking thiamine and binding to the TPP riboswitch. Certain strains of *Bacillus subtilis *have evolved resistance to pyrithiamine by at least two distinct means. Firstly, via mutations to the ligand-binding aptameric region of the riboswitch, and secondly by overexpressing a thiaminase enzyme (Sudarsan et al., 2005). It may therefore be prudent to select target riboswitch classes which regulate multiple genes or operons in a given genome, rather than a single gene or operon. Furthermore, suitable candidates should exert important gene regulatory functions for cellular survival, to ensure a lower rate of mutational resistance.

## FUTURE PERSPECTIVE

The RNA-based strategies outlined above are at varying stages of progress toward potential therapeutic utility. A major challenge in the development of any antibacterial drug is in delivery across the cell wall, in particular the peptidoglycan layer of gram positive bacteria, to reach the cytoplasm. Recently, penicillin has been shown to increase the uptake efficiency of antisense PMOs, likely through the inhibition of peptidoglycan synthesis (McLeod and Simmonds, 2013). Thus, systemic searches of compounds known to disrupt the integrity of the cell wall may lead to the identification of suitable co-delivery agents.

The development of additional methods for delivery is also likely to expedite the drug development process. One can envisage a scenario in which drugs targeting homologs of the same gene could be administered differently depending upon the infectious agent. For example, although not extensively researched, liposomes have been effectively used to deliver antisense PMOs to the gram-positive methicillin-resistant *S. aureus* (MRSA; Meng et al., 2009). Liposome delivery has the conceptual advantage of avoiding potential resistance issues related to protein transport such as those described above, and may be a useful means to augment the uptake of LNA/DNA hybrid molecules, which cannot be conjugated to proteins.

Increasing the potency of new drug candidates, such that they may be used at lower effective concentrations, will be another important step toward their transfer to the clinical environment. In this regard, atsRNAs may represent a more attractive blueprint than asRNAs, given the apparently increased efficacy of the former. Additive antimicrobial effects by silencing multiple important genes have been shown (McKinney et al., 2001), however, synergistic effects have not yet been demonstrated. Synergism in drug interactions can lead to dramatically improved clinical outcomes (Chou, 2006), and synergism between protein- and RNA-level inhibitors is known (Dryselius et al., 2005). Database searches reveal no shortage of interacting pairs of genes that may be adapted for focused therapeutic designs (Yeh et al., 2009).

These aspects notwithstanding, there is no theoretical reason that RNA-based antibacterial therapies should not continue to progress toward the clinical sphere. *In vivo* work has shown their utility in treating both localized (Sawyer et al., 2013) and systemic (Meng et al., 2009) infections, as proof of concept. Indeed, their therapeutic development ought to be an inevitability, given that antisense strategies have been used in clinical practice for over a decade to treat viral infections (de Smet et al., 1999). Moreover, a number of antisense-based treatments of non-bacterial diseases – such as Duchenne muscular dystrophy – are currently in clinical trials (see, for example, http://www.sareptatherapeutics.com/). With the present rate of advance, it may be anticipated that sufficient knowledge of design and delivery principles will, within the next decade, lead to the development of antibacterial compounds suitable for clinical trial.

## Conflict of Interest Statement

The authors declare that the research was conducted in the absence of any commercial or financial relationships that could be construed as a potential conflict of interest.
